# Wild rodents seed choice is relevant for sustainable agriculture

**DOI:** 10.1038/s41598-024-67057-y

**Published:** 2024-07-10

**Authors:** Yang Peng, Zhenbang Hu, Wen Dong, Xiaodong Wu, Chunyan Liu, Rongsheng Zhu, Jinhui Wang, Mingliang Yang, Zhaoming Qi, Ying Zhao, Jianan Zou, Xiaoxia Wu, Yingdong Bi, Limin Hu, Pascal Ratet, Qingshan Chen, Dawei Xin

**Affiliations:** 1 National Key Laboratory of Smart Farm Technologies and Systems, Key Laboratory of Soybean Biology in Chinese Ministry of Education, College of Agriculture, Northeast Agricultural University, Harbin, 150030 People’s Republic of China; 2 Université Paris-Saclay, CNRS, INRAE, Univ Evry, Institute of Plant Sciences Paris-Saclay (IPS2), 91190 Gif sur Yvette, France; 3 Institute of Plant Sciences Paris-Saclay (IPS2), Université de Paris, 91190 Gif sur Yvette, France; 4 Institute of Crop Cultivation and Tillage, Heilongjiang Academy of Agricultural Sciences, Harbin, 150028 Heilongjiang, China

**Keywords:** Plant sciences, Ecology

## Abstract

Mitigating pre-harvest sprouting (PHS) and post-harvest food loss (PHFL) is essential for enhancing food securrity. To reduce food loss, the use of plant derived specialized metabolites can represent a good approach to develop a more eco-friendly agriculture. Here, we have discovered that soybean seeds hidden underground during winter by *Tscherskia triton* and *Apodemus agrarius* during winter possess a higher concentration of volatile organic compounds (VOCs) compared to those remaining exposed in fields. This selection by rodents suggests that among the identified volatiles, 3-FurAldehyde (Fur) and (E)-2-Heptenal (eHep) effectively inhibit the growth of plant pathogens such as *Aspergillus flavus, Alternaria alternata, Fusarium solani* and *Pseudomonas syringae*. Additionally, compounds such as Camphene (Cam), 3-FurAldehyde, and (E)-2-Heptenal, suppress the germination of seeds in crops including soybean, rice, maize, and wheat. Importantly, some of these VOCs also prevent rice seeds from pre-harvest sprouting. Consequently, our findings offer straightforward and practical approaches to seed protection and the reduction of PHS and PHFL, indicating potential new pathways for breeding, and reducing both PHS and pesticide usage in agriculture.

## Introduction

Pre-harvest sprouting (PHS) and post-harvest food losses (PHFL) significantly undermine food security^1–3^. Seeds typically undergo a period of diminished germination capability known as dormancy, which is most pronounced at physiological maturity and diminishes thereafter^4,5^.

Postharvest loss (PHL) of food crops, which occurs during or following harvest, results in significant food wastage and squanders the resources invested in their production and distribution^6^. Annually, crop yield losses attributable to pathogens and pests alone are estimated at US$220 billion^7^. Seeds often harbor a variety of microorganisms, particularly fungi, which pose considerable sanitary challenges not only to the seeds and growing crops but also to human and animal health^8,9^. The deleterious impacts of seed-associated fungi include facilitating the spread of plant diseases, degrading seed quality and longevity, and producing mycotoxins that are highly toxic when consumed by humans and animals.

For instance, pathogens such as *Alternaria alternata, Fusarium solani,* and *Pseudomonas syringae* negatively impact crop growth and yields. Moreover, agricultural crops colonized by the pervasive saprotrophic fungus *Aspergillus* *flavus* may be tainted with aflatoxin, a potent mycotoxin that poses serious threats to food security and the agricultural economy^10^.

Cleaning grains going into storage to remove lightweight and broken kernels or seeds as well as foreign material and fines is one of the main approaches to prevent pathogen infection of stored seeds. Another way is to store the dry grains under safe low moisture atmosphere as quickly as possible^11^. Gaseous ozone and physical treatments have interesting potential for grain safety management, but are presently uncommon because of high costs, apart from the need of refrigeration for high quality products^12,13^. VOCs produced by seeds to prevent pathogen infection may represent an alternative functional method for protection, that can additionally represent an environment-friendly choice ^14^.

To survive the winter, rodents must store sufficient food underground. This stored food also encounters risks of PHS and PHFL. In the northeast of China (Harbin), where winters last approximately six months, it is hypothesized that rodents may select soybean seeds with specific traits that minimize losses in their burrows. Good conditions for soil digging by the rodents are when the temperature is above 0 ℃ and the weather sunny and dry during fall. After this period the temperatures reach 0 ℃ or below and the soil become too cold and hard for digging the burrow. The decision to prepare the burrow and how long it requires might result from the capacity of the rodents to estimate how long they need to complete a task^15^. Temperature and humidity conditions in the burrow should also help reducing the PHS and PHFL in order to sustain rodent food for the long winter.

In addition, the animals may choose the most appropriated seeds for long term storage. Indeed, rodent live in a rich olfactory world, able to detect and recognize immense numbers of odors and distinguish minute differences in complex mixtures^16,17^. We analyzed the composition of the soybean seeds hidden in the burrow (rodent-hidden seeds, RHS) and compared it to soybean seeds left on the plant in the field (rodent-unhidden seeds, RUS) in a three year experiment using seed materials from Xiangyang Farm in 2019(2019XY), Xiangyang Farm in 2021(2021XY), and Minzhu Experimental Station in 2021(2021MZ). This study aimed to provide a scientific base for understanding the function of seed VOCs in preventing the pathogen infections and in their effects on seed germination.

## Results

### Period of rodent cached seeds

**Figure 1:**
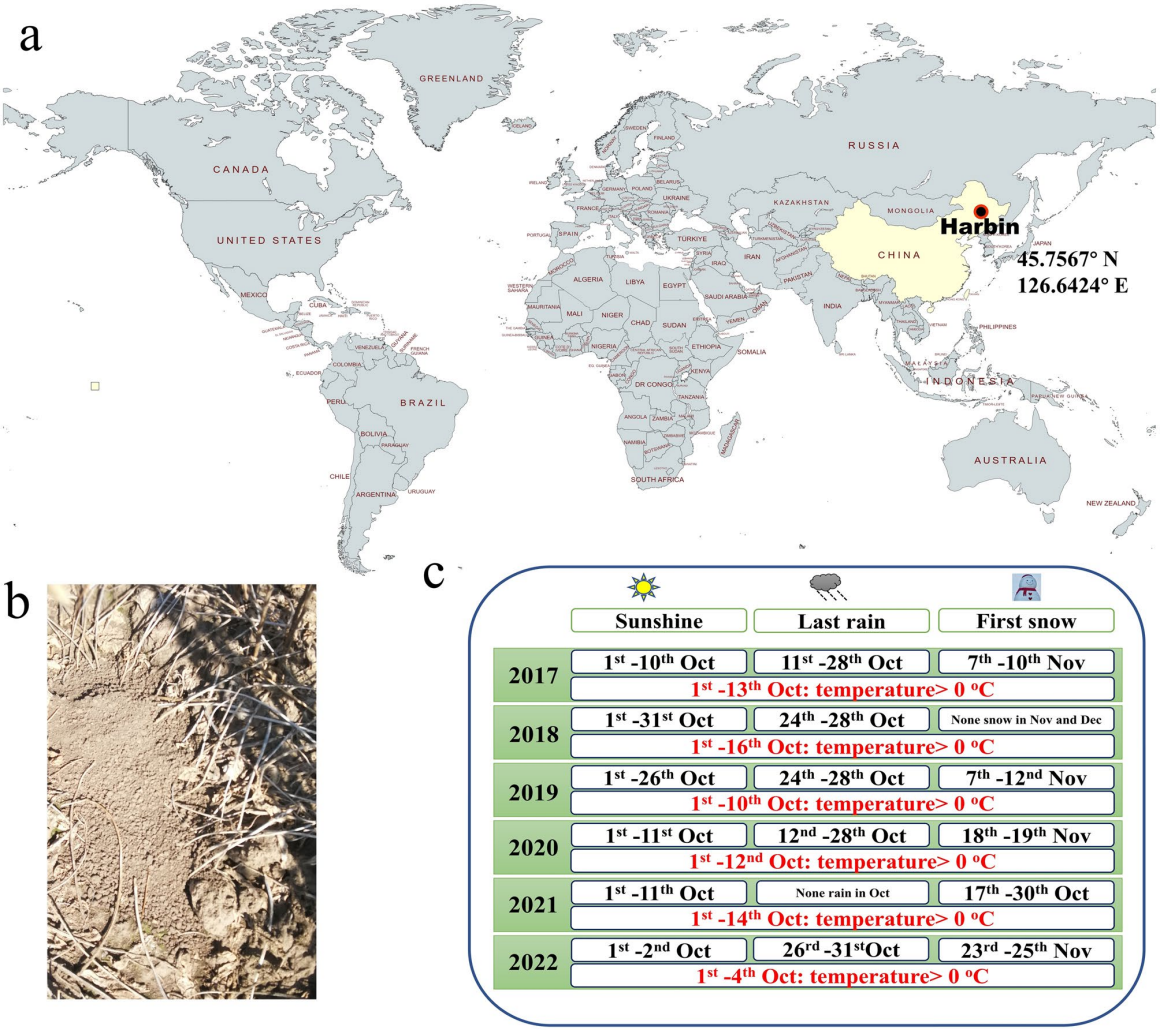
Dates to dig the rodent burrow. (**a**) Location of Harbin in the world. (The map was drawn using MapMaker 2.0 National Geographic Headquarters, Washington, DC https://mapmaker.nationalgeographic.org/). (**b**) Sealed rodent burrow in a soybean field. (**c**) Weather conditions in Harbin. The sunny days, rainy days and first snow fall for the October–November period are indicated.

Heilongjiang province is the largest soybean planting area in China. In Harbin (45.7567° N, 126.6424° E) (Fig. 1a), soybeans are typically harvested from October 1st to 25th, prior to the Frost Descent, one of China’s 24 Solar Terms. At this time, most soybean cultivar have reached the mature stage and are suitable for harvest. The Frost Descent of China’s 24 Solar Terms season is characterized by colder weather in the morning and evening, hotter weather at noon, a big temperature difference between day and night, and obvious autumn dryness. On average across the country, it is the time of year with the greatest difference between day and night temperatures. After entering the frosty season, the cold air activity affecting China becomes more and more frequent, and the temperature difference between day and night increases rapidly. Around the beginning of winter, stronger windy and cooling weather often occurs, and some places cross over into winter in a relatively short period of time. All the soybean should be harvested before the first frosts.

Early October marks the phase when most soybean seeds have matured, aligning with the time when field rodents (*Tscherskia triton* and *Apodemus agrarius*) finish preparing their over-wintering burrows (OWB) (Fig. 1b). We found that the over-wintering burrows appeared in the field and were completed during 7th–14th Oct at sunshine conditions (Fig. 1c and Extended Data Fig. 1). RHS and RUS seeds were then harvested the 10th or 11th of October in year 2019 and 2021 (see “Materials and methods”) for analysis. In 2019, one burrow was selected, and in 2021 two burrows were selected. In each of these three burrows, about 5 kg seeds were present and this amount was enough for analysis.

### Protein, oil and tannin composition of the burrowed seeds

**Figure 2:**
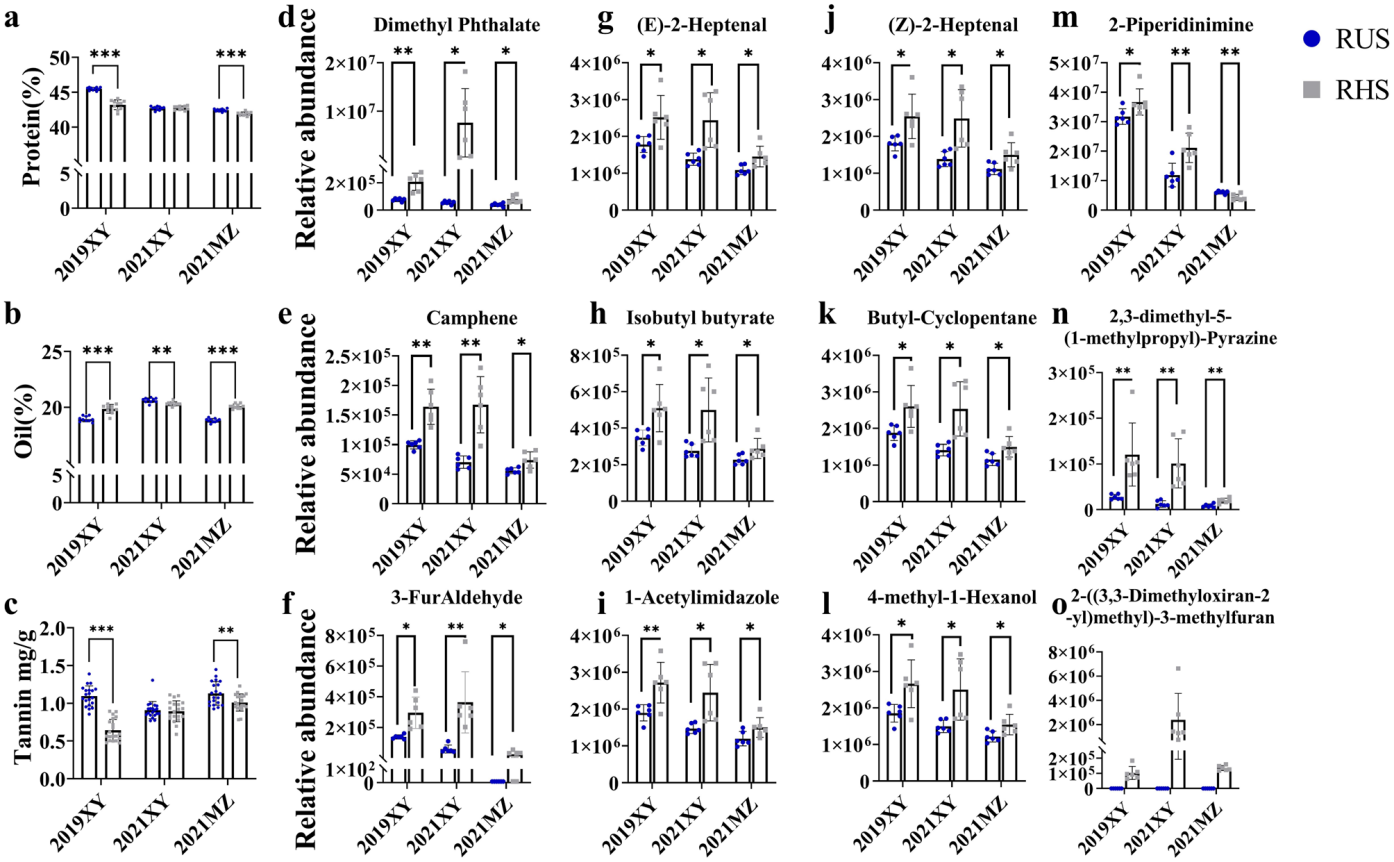
Composition analysis of rodent-hidden seeds (RHS, gray squares) and rodent-unhidden seeds (RUS, blue circles). (**a**) Protein content in RHS and RUS. n = 20 (**b**), oil content in RHS and RUS. n = 20 (**c**), tannin content in RHS and RUS. n = 20 (**d–o**), the identified 12 volatile organic compounds in RHS and RUS. n = 6 (The statistical student’s t-test with calculated p-value less than significant level (α) = 0.05 was considered; * ≤ 0.05; ** ≤ 0.01; *** ≤ 0.001). Details are provided in Supplemental Materials and Methods. 2019Xiangyang Farm soybean (2019XY), 2021Xiangyang Farm soybean (2021XY), 2021Minzhu Experimental Station soybean (2021MZ).

The protein, oil and fatty acid contents showed no significant or consistent differences between RHS and RUS seeds across the three sample sets (times and locations) analyzed (Fig. 2a, b, Extended Data Fig. 2 and Extended Data Table 1). Our findings do not align with previous studies suggesting rodents' preference for nutrient-rich seeds^16^, but may reflect the inherently high protein and oil composition of soybean seeds, unlike those from the *Aizoaceae*, *Asteraceae*, *Poaceae*, and *Polygonaceae* families^17^.

However, tannin levels exhibited significant differences (Fig. 2c, Extended Data Fig. 3, and Extended Data Table 1). The lower tannin content in RHS seeds correlates with the known preference of birds and other animals for seeds with less tannin for future hoarding^18^.

### Volatile compounds enriched in RHS can inhibit pathogens growth

**Figure 3:**
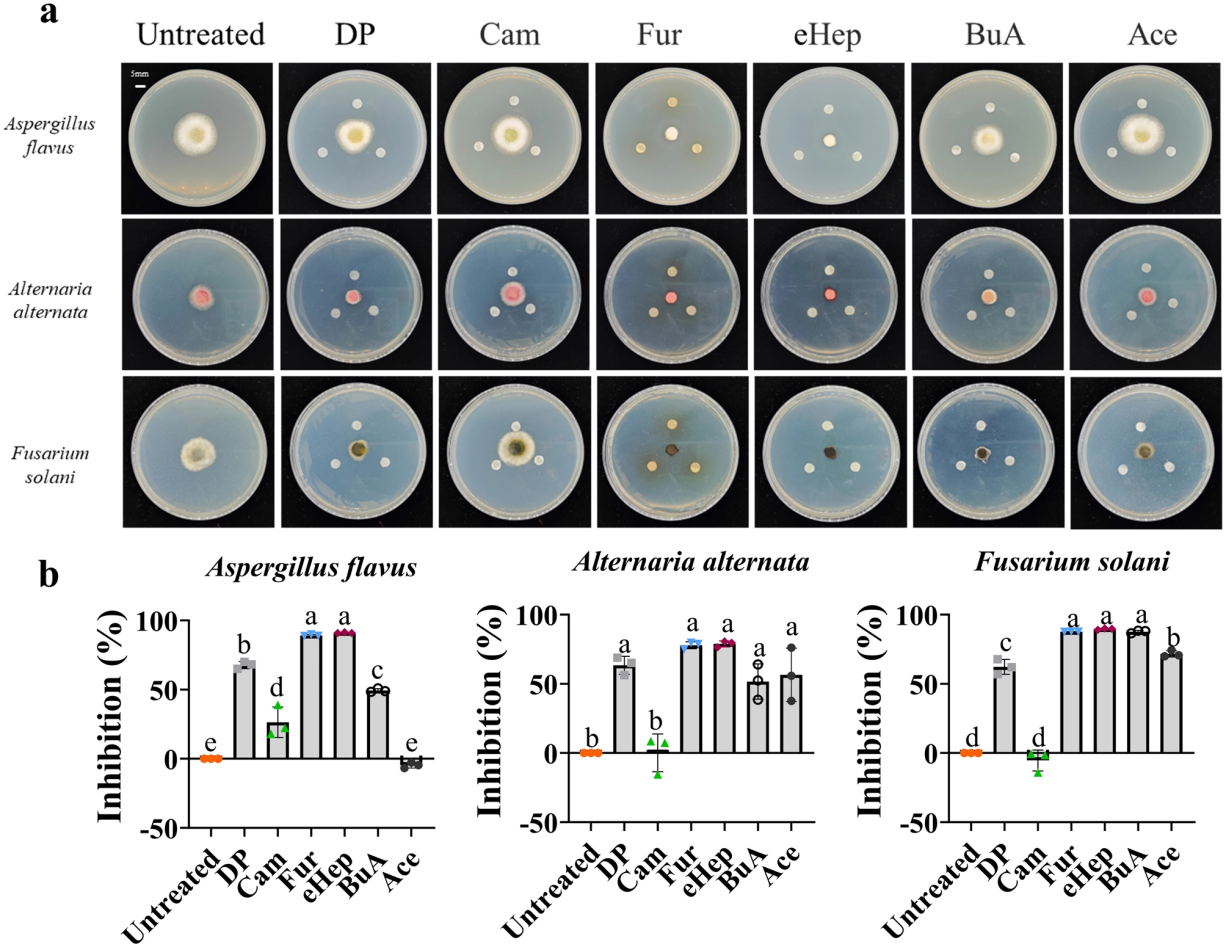
Inhibition analysis of volatile organic compounds to fungus pathogen. (**a**) Halo test showing the antifungal activity of six volatile organic compounds. (**b**) Inhibition capacity of six volatile organic compounds to *Aspergillus flavus*, *Alternaria alternata*, *Fusarium solani*. n = 3 (One-way ANOVA with Tukey’s multiple comparisons test Columns bearing different letters are significantly different.) Dimethyl phthalate (DP); Camphene (Cam); 3-FurAldehyde (Fur); (E)-2-Heptenal (eHep); Isobutyl butyrate (BuA); 1-Acetylimidazole (Ace).

We then examined the volatile organic compounds (VOCs) in RHS and RUS seeds. Out of 400 compounds detected (Extended Data Table 2), 12 showed varying levels between RHS and RUS seeds (Fig. 2d–o). Only six out of twelve differentially produced VOCs were studied because these were the only ones commercially available. As the RHS need to be stored in rodent burrows for at least six months, we tested these six VOCs for their ability to inhibit fungal and bacterial pathogen growth. Using halo analysis^19^, five VOCs demonstrated significant inhibition of pathogen growth. These VOCs include Dimethyl phthalate (DP), 3-FurAldehyde (Fur), (E)-2-Heptenal (eHep), Isobutyl butyrate (BuA), and 1-Acetylimidazole (Ace) (Fig. 3). The growth of *Aspergillus flavus*, *Alternaria alternata*, *Fusarium solani*, and *Fusarium tricinctum* (Fig. 3 and Extended Data Fig. 4) was significantly reduced by these VOCs. Additionally, the growth of bacteria such as *Escherichia coli* DH5α, *Pseudomonas syringae*, *Agrobacterium tumefaciens* GV3101, and *Sinorhizobium fredii* HH103 was also inhibited by some of these VOCs (Extended Data Fig. 4). This indicates that the VOCs produced by these soybean seeds confer to them the capacity to resist to pathogens.

### Inhibition of seed germination and pre-harvest sprouting

**Figure 4:**
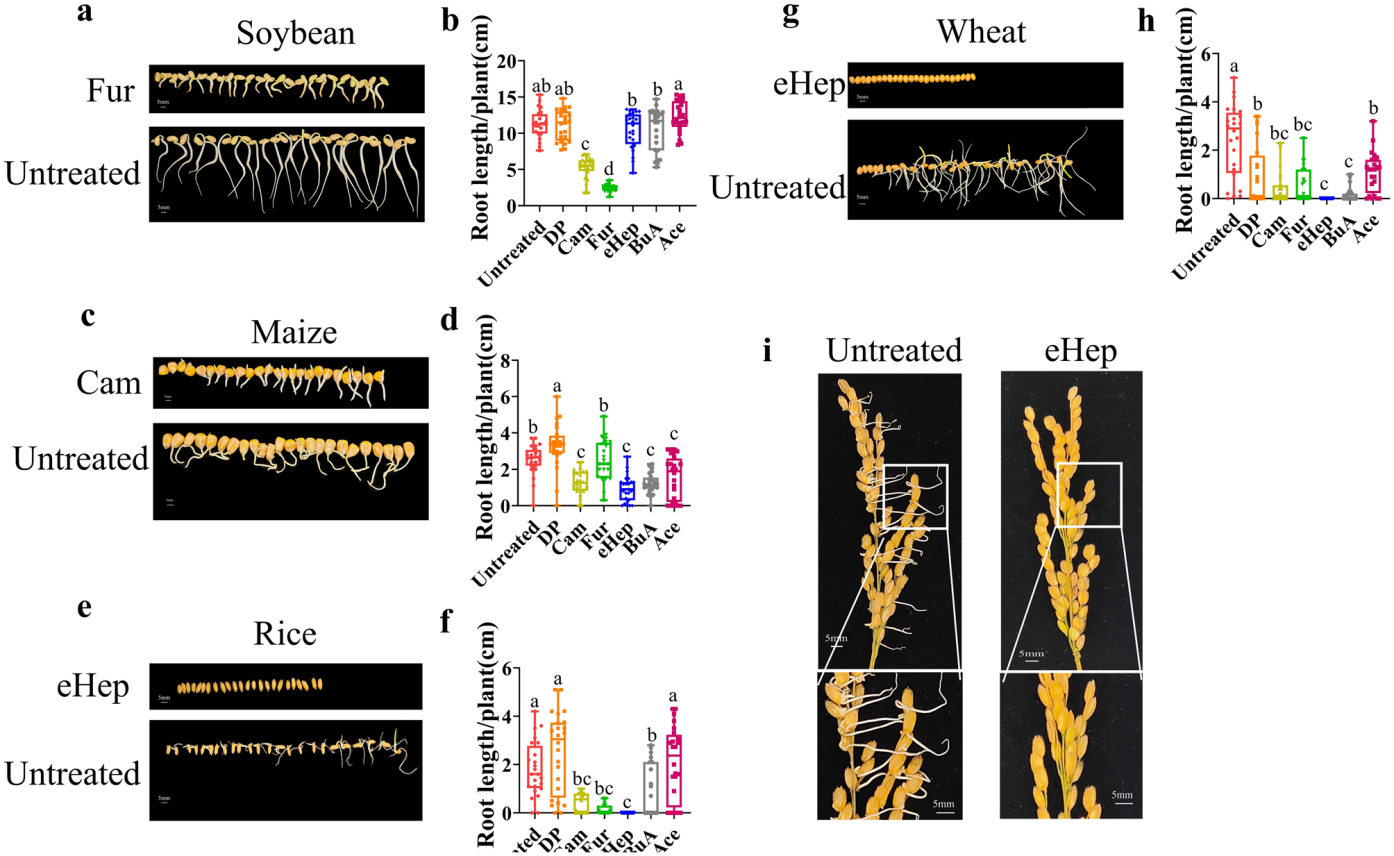
Inhibition of seed germination. (**a**) Soybean seed germination inhibition by 3-FurAldehyde (Fur). (**b**) Root length growth of soybean treated by volatile organic compounds. n = 24 (**c**), maize seed germination inhibition by Camphene (Cam). (**d**) Root length growth of maize treated by volatile organic compounds. n = 24 (**e**), rice seed germination inhibition by (E)-2-Heptenal (eHep). (**f**) Root length growth of rice treated by volatile organic compounds. n = 24 g, Wheat seed germination inhibition by (E)-2-Heptenal (eHep). (**h**) Root length growth of wheat treated by volatile organic compounds. n = 24 (**i**), rice pre-harvest sprouting inhibited by (E)-2-Heptenal (eHep). n = 3 (One-way ANOVA with Tukey’s multiple comparisons test Columns bearing different letters are significantly different.) Dimethyl phthalate (DP); Camphene (Cam); 3-FurAldehyde (Fur); (E)-2-Heptenal (eHep); Isobutyl butyrate (BuA); 1-Acetylimidazole (Ace).

We hypothesized that these VOCs could affect crop seed germination and tested the impact of six VOCs (see “Materials and Methods” section) on the germination of two soybean genotypes, as well as maize, rice, and wheat. We identified four volatiles that inhibited seed germination across these species (Fig. 4a–h and Extended Data Fig. 5–9). Besides germination, the VOCs significantly reduced hypocotyl elongation. 3-Furaldehyde inhibited soybean hypocotyl elongation (Fig. 4a and b), while camphene was the most potent inhibitor of maize radicle and coleoptile elongation (Fig. 4c and d). Rice seed germination and hypocotyl elongation were completely inhibited by (E)-2-Heptenal (Fig. 4e and f). Wheat seeds, which were particularly sensitive to (E)-2-Heptenal, experienced complete inhibition of both germination and hypocotyl elongation (Fig. 4g and h). Pre-harvest sprouting in rice was also inhibited by both (E)-2-Heptenal and 3-FurAldehyde (Fig. 4i and Extended Data Fig. 10). These findings suggest that the selection of seeds by rodents is influenced by their suitability for long-term storage.

## Discussion

Our study analyzed the VOCs produced by soybean seeds hidden by *T. triton* and *A.* *agrarius* in burrows for long-term storage (RHS) and compared them with the RUS around the rodent burrow left on the plants in the field in years 2019 and 2021. At the end of autumn, wild rodents in the field dig burrows to store seeds for overwintering (Extended Data Fig. 1). We observed that these burrows were created in the first two weeks of October, a time characterized by dry soil conditions ideal for digging (Fig. 1c). The seeds stored in these burrows were excavated during the second week of October, prompting us to hypothesize differences in composition between the RHS and RUS. Although rodents are known to select nutritionally rich seeds^17^, our analysis of RHS and RUS seed quality revealed no consistent differences across the studied years (Fig. 2, Extended Data Fig. 3, and Extended Data Table 1), probably because the fact that the excavation site was consistently planted with the Heinong83 soybean cultivar.

However, our findings confirm our hypothesis that the VOC composition of seeds stored for overwintering differed from those left on the field. In various plant species, maternal factors such as the position of the inflorescence on the mother plant or the position of seeds within the inflorescence or pod can significantly affect seed size, composition, and maturation stage^20–22^. Long-term selection experiments with maize showed that lower and higher protein and oil containing seeds do appear in each generation^23^. In nature, animals significantly contribute to plant pollination and seed dispersal. Utilizing their olfactory abilities, for instance, mice can excavate newly sown wheat seeds^21^, targeting those rich in nutritionally valuable oils. Rodents like *T. triton* and *A.* *agrarius* may also have developed the ability to recognize VOCs emitted by seeds, which are indicative of seed dormancy and pathogen resistance. As soybean seeds are rich in protein and oil, their selection by rodents might not depend on the amount of these compounds but on their quality and composition. More experiments are required to determine if the protein and oil contents or compositions are factors affecting the rodent choice.

*A. alternata* was the predominant fungal species isolated from sorghum, rice, soybean seeds, and freshly harvested wheat^24^. *F. tricinctum* and *F. solani* are known to cause various plant diseases globally, especially in temperate regions^25,26^. Our results demonstrate that the Fur and eHep VOCs in stored seeds inhibit the growth of *F. tricinctum* and *F. solani* (Fig. 3a and Extended Data Fig. 4). Utilizing a cocktail of Fur and eHep could mitigate and prevent the growth of these fungi*.* Similarly, the inhibition of *Aspergillus* *flavus* by these VOCs, could significantly reduce the risk of aflatoxin contamination in food and feed. Although chemical control has been employed to manage pathogenic fungal infections in crops, its effectiveness in controlling diseases and reducing mycotoxin levels in major crops like wheat, rice, maize, and soybean is inconsistent. Conversely, chemical treatments may substantially reduce fungal infections in fungicide-treated plots post-application^27^. Seed derived VOCs not only can prevent fungal infections but also halt the propagation of seed-borne diseases. Floral volatiles act as attractants for species-specific pollinators, while volatiles from vegetative parts, particularly those released after herbivore damage, help protect plants by deterring herbivores and attracting their predators^28^. We identified five out of twelve VOCs from seeds that exhibit diverse functions in preventing the growth of pathogenic fungi and bacteria. As it is very difficult to assess the absolute concentrations of VOCs in the seeds and it is also difficult to keep a pre-established and constant concentration of VOCs in the air of the Petri dishes, we placed 10 μL of each VOC on a sterile filter paper discs at the center of the culture medium to analyze the VOC effects on the growth of the pathogens. For future experiments, it will be important to better estimate the volatile natural concentrations in order to better understand their action^29^. These findings suggest that volatiles emitted from seeds could serve as an additional defense strategy against pathogens.

Identifying the resistant genes that underpin crop resistance to diseases is a traditional approach to mitigating disease-related yield and seed quality losses. However, this method requires extensive germplasm selection and genetic analysis. The absence of genomic information or complex genomic structures can also hinder breeding processes^30^. Rodent selection of seeds with specific VOCs composition for overwinter storage in burrows at a special period in each year, suggests a specific synthesis of these VOCs at late stage of seed development. The identification of genes responsible for the synthesis in seeds of these VOC involved in resistance against pathogens, would give interesting tools for breeding and the development of an eco-friendly agriculture.

In addition to inhibiting pathogens growth, we found that the VOCs can also suppress seed germination. For rodents, the ability to inhibit seed germination is crucial for long-term storage in burrows. The distinct germination inhibition capacity of (E)-2-Heptenal and 3-FurAldehyde supports the notion that plants might also utilize these VOCs to regulate seed dormancy. We observed that some seeds germinated within the burrow (Extended Fig. 1I), indicating that plant-derived VOCs do not have long-term negative effects on seed germination, unlike artificial chemicals^31^. In addition, this might suggest that the concentration of VOCs close to the entrance of burrow might be lower. VOCs demonstrate varying inhibition capacities on specific crop seeds (Extended Data Figs. 5–9), suggesting a cocktail effect of VOCs.

This work suggests that the content and composition of VOCs in seeds have the potential to reduce main crop PHS and PHFL, and could help decrease the use of synthetic pesticides in agriculture. The identification of genes controlling the VOC production in seeds might help developing crops with better resistance to pathogens. The function of VOCs in controlling seed germination might also supply new hints for the study of seed dormancy.

## Materials and methods

### Soybean seeds isolation

Rodent-hidden and rodent-unhidden seeds were isolated from two farm approximately 15 km east of Harbin, including Xiangyang Farm (XY) (45°84′ N, 126º85′E) and Minzhu Experimental Station (MZ) (45°77′ N, 126°91′ E). The dates for excavating the cached seeds in the rodent burrows were October 10, 2019 (XY), and October 11, 2021 (XY, MZ), respectively. Fields planted exclusively with the soybean cultivar Heinong83 were chosen for locating the rodent burrows^32^.

### Soybean quality

Unhidden seeds located around the rodent burrows were selected as controls. Protein and oil contents were analyzed using a near-infrared spectrometer with six repeats (FOSS, Infratec™ 1241)^33^. The tannin content of the seed coat and cotyledon of the soybean seeds was determined using the Phosphomolybdic acid method, kit (Suzhou Grace Biotechnology Co., Ltd., Suzhou, China)^34^.

### Fatty acids (FAs) extraction and determination

The 18 essential FAs (Butryic acid, Capric acid, Lauric acid, Myristic acid, Palmitic acid, Palmitoleic acid, Heptadecanoic acid, Stcaric acid, Oleic acid, Linoleic acid, α-Linolenic acid, Arachidic acid, Eicosenoic acid, Henicosanoic acid, Behenic acid, Lignoceric acid, Docosahexaenoic acid, and Nervonic acid) were derivatized to their methyl esters and analyzed using gas chromatography^35^. Briefly, 1 g of seeds from each sample was finely ground using a Sample Preparation Mill. Then, 50 mg of this powder was weighed using an analytical balance, dissolved in ethanol to a final volume of 25 mL, from which 1 mL was taken and added to a 15 mL centrifuge tube. This was followed by the addition of 2 mL of 5% methanol hydrochloride solution, 3 mL of chloroform methanol solution (volume ratio 1:1), and 100 μL of Nonadecanoic acid methyl ester as an internal standard. The mixture was incubated for 1 h at 85 °C. After incubation, 1 mL of n-hexane was added and the mixture was shaken for 2 min and left to stand for 1 h. The upper layer of clear liquid (100 μL) was then taken, diluted with n-hexane to 1 mL, and passed through a 0.45 μm filter membrane for analysis by a GC–MS system. The supernatant was assayed using a Trace1310 gas chromatograph (ThermoFisher, U.S.) equipped with a TG-5MS column (30 m × 0.25 mm × 0.25 μm). The temperature program began at 80 °C for 1 min, increased to 200 °C at 10 °C/min, to 250 °C at 5 °C/min, to 270 °C at 2 °C/min, and was held at 270 °C for 3 min. The carrier gas was helium, flowing at 1.2 mL/min, and 1 μL of each sample was injected. The ionization potential of the mass-selective detector was 70 eV, and the mass spectrometry was operated at 280 °C. The solvent delay time was 5 min, and the full-scan mode ranged from 30 to 400 amu. Data were normalized by calculating the peak area ratios of each peak to the internal standard^36^.

### Identification of volatile compounds

Head-space solid-phase microextraction (HS-SPME) was utilized to collect volatiles from 500 mg of seed, which were absorbed by a 120 µm divinylbenzene/carboxen/polydimethylsiloxane fiber (Agilent Technologies, CA, USA) for 15 min at 60 °C. The collected volatiles were then analyzed by gas chromatography-mass spectrometry (GC–MS) using an Agilent 8890 GC and Agilent 5977B MS, equipped with a DB-5MS capillary column (30 m × 0.25 mm × 0.25 μm; Agilent Technologies). Helium served as the carrier gas at a linear velocity of 1.2 mL/min^37^.

### Pathogen inhibition analysis

Following the method of Lim et al., we used three 0.6 cm (diameter) sterile filter paper discs containing 10 μL VOCs and put the sterile filter paper discs individually around the center of the culture medium. Dimethyl phthalate (99%, D806666), Camphene (96%, C804852), 3-FurAldehyde (98%, F810185), (E)-2-Heptenal (95%, H838703), Isobutyl butyrate (98%, I858262), and 1-Acetylimidazole (98%, A800030) were obtained from Macklin. Mycelia from *Aspergillus flavus*, *Fusarium oxysporum*, *Fusarium trifidum*, and *Alternaria alternata* were placed at the center of PDA culture medium (Potato extract, 1L d-glucose, 20 g/L Agar, 20 g/L) individually. For bacterial growth inhibition, 100 µL of bacterial suspension (OD_600_ = 0.2) of *Escherichia coli* DH5α*, Agrobacterium tumefaciens* GV3101*, Sinorhizobium fredii* HH103, and *Pseudomonas syringae* was plated on Luria–Bertani media (Tryptone, 10 g/L Yeast Extract, 5 g/L NaCl, 10 g/L Agar, 15 g/L), TY Medium (Tryptone, 10 g/L Yeast Extract, 5 g/L CaCl_2_, 10 mmol/L Agar, 15 g/L) and Nutrient Yeast Glycerol media (Tryptone, 5 g/L Yeast Extract, 3 g/L Glycerin, 20 mL/L Agar, 15 g/L). The sterile discs were placed around the center of the plate before bacterial growth. *Aspergillus flavus*, *Fusarium oxysporum*, *Fusarium trifidum*, and *Alternaria alternata* were placed in an incubator at 28 °C for 72 h, *Escherichia coli* DH5α was placed in an incubator at 37 °C for 24 h, and *Agrobacterium tumefaciens* GV3101*, Sinorhizobium fredii* HH103 and *Pseudomonas syringae* were placed at 28 °C for 48 h, with three replicates set for each species before measuring the zone of inhibition for bacteria and fungi ^19^.

### Seed germination assays

Germination was measured according to previously established methods^38^. Twenty-four seeds each of rice (Dongnong428, DN428), maize (Dongnong285, DN285), wheat (Chinese Spring, CS), soybean (Dongnong50, DN50; Suinong14, SN14) were putted on a round Petri dish 12 cm in diameter with sterilized filter papers, 12 mL sterilized distilled water were used to keep the humidity for maize and soybean, 7 mL sterilized distilled water were used to keep the humidity for rice and wheat. A 250 μL PCR tube with 100 μL volatile compound (100%) was putted in the center and a hole was made to allow the volatile to evaporate. In this experiment the volume of VOC did not change much by the end of the experiment, indicating that the compounds diffused slowly. For each germination assay, at least three panicles were treated and incubated at 28 °C in a growth chamber. For each germination assay, at least 24 seeds were used for each dish replicate and 3 technical replicates were treated and incubated at 25 °C in a growth chamber. The number of germinated seeds was counted for 4 days and daily until most of the seeds germinated.

For pre-harvest sprouting germination assays, freshly harvested Kitaake panicles were soaked in H_2_O. At least three panicles were treated per assay and incubated at 28 °C in a growth chamber. Panicles were transferred to fresh water or solution daily. The number of germinated seeds was counted daily for four days until most had germinated.

Germination was defined as the length of coleoptiles equaling one-half of the length of seeds^39^.

### Data analysis

Statistical analysis was performed using Statistical Product Service Solutions 27 (SPSS 27). The statistical student’s t-test was used for the composition analysis of rodent-hidden seeds (RHS) and rodent-unhidden seeds (RUS) include the oil, protein, tannin, volatile organic compounds and fatty acids composition. (p values less than 0.05 were considered statistically significant.) Repeated measures one-way ANOVA with Tukey's multiple comparisons test was used for the longitudinal analysis of the inhibition analysis of volatile organic compounds to fungus pathogen and the inhibition of seed germination. (Different letters are significantly different).

### Ethics approval and consent to participate

Experimental research and field studies on plants (either cultivated or wild), including the collection of plant material, must comply with relevant institutional, national, and international guidelines and legislation. The collecting of these plant materials complies with the IUCN Policy Statement on Research Involving Species at Risk of Extinction and is allowed by the Convention on the Trade in Endangered Species of Wild Fauna and Flora.

### Supplementary Information

Supplementary Figures.

Supplementary Table 1.

Supplementary Table 2.

## Data Availability

The datasets used and/or analysed during the current study available from the corresponding author on reasonable request.
