# Supplementary material for: Wild rodents seed choice is relevant for sustainable agriculture
Source: Sci Rep. 2024 Jul 10;14:15994. doi: 10.1038/s41598-024-67057-y (PMC11237120; doi:10.1038/s41598-024-67057-y)
Supplement: Supplementary file 1 — Supplementary Figures. [file 41598_2024_67057_MOESM1_ESM.docx]

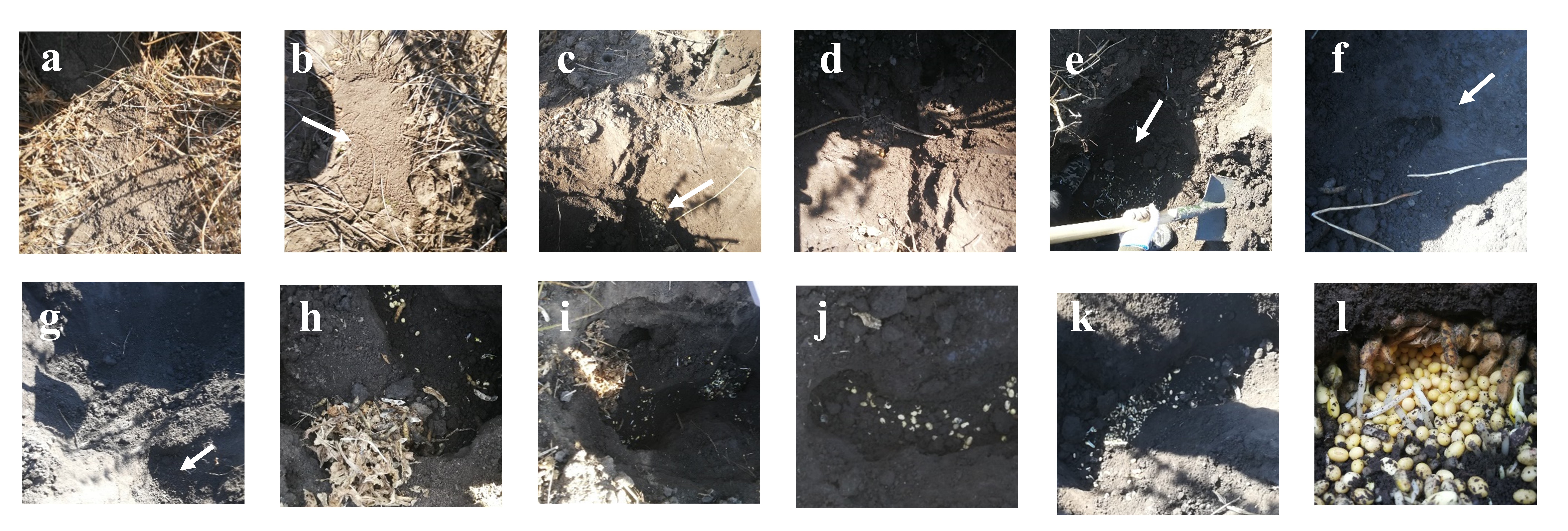


Extended Data Fig. 1 Dug field rodent burrow for the cached soybean seeds. **a** and **b**, Excavated soils of field rodent burrow, the soil were covered on the vents of burrow. **c-g**, Process of digging a field mouse hole. **h-l**, Soybean hoarding cell of field rodent burrow.





Extended Data Fig. 2 Fatty acids composition analysis of rodent-hidden seeds (RHS, gray squares) and rodent-unhidden seeds (RUS, blue circles). **a-r**, 18 fatty acids were detected. Butryic acid (BA), Capric acid (CA), Lauric acid (LaA), Myristic acid (MA), Palmitic acid (PA), Palmitoleic acid (PaA), Heptadecanoic acid (HepA), Stcaric acid (SA), Oleic acid (OA), Linoleic acid (LA), α-Linolenic acid (ALA), Arachidic acid (AA), Eicosenoic acid (EA), Henicosanoic acid (HeA), Behenic acid (BeA), Lignoceric acid (LigA), Docosahexaenoic acid (DHA), Nervonic acid (NA). 2019 Xiangyang Farm soybean-Filial generation (19XY-F­_1_), 2021 Xiangyang Farm soybean (21XY), 2021 Minzhu Experimental Station soybean (21MZ). n =3 (The statistical student’s t-test with calculated p-value less than significant level (α) = 0.05 was considered; * ≤0.05; **≤0.01; ***≤0.001).


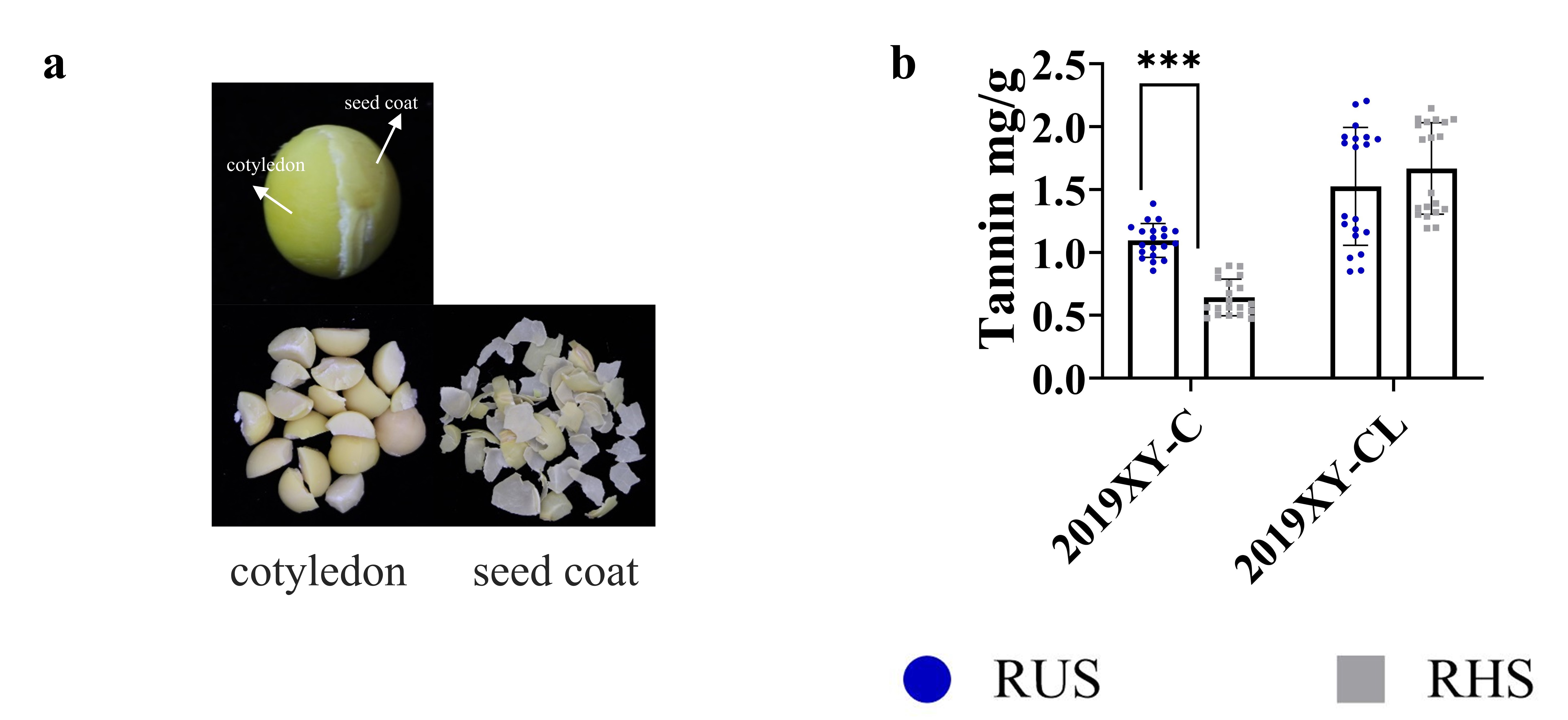


Extended Data Fig. 3 Tannin content in the seed coat and cotyledon. **a**, Soybeans cotyledon and seed coat. **b**, Tannin content in the 2019Xiangyang Farm soybean seed coat (2019XY-C) and 2019Xiangyang Farm soybean cotyledon (2019XY-CL). n =20 (The statistical student’s t-test with calculated p-value less than significant level (α) = 0.05 was considered; * ≤0.05; **≤0.01; ***≤0.001).





Extended Data Fig. 4 Volatile compounds (VOCs) inhibit the growth of fungi and bacteria. **a**, Inhibition of VOCs on *Fusarium tricinctum*, *Pseudomonas syringae*, *Escherichia coli* DH5α, *Agrobacterium tumefaciens* GV3101, *Sinorhizobium fredii*. The inhibition is visualized by halos around the filter paper. Details are provided in Supplemental Materials and Methods. **b**, Statistics of the inhibition by the VOCs on *Fusarium tricinctum*, *Pseudomonas syringae*, *Escherichia coli* DH5α, *Agrobacterium tumefaciens* GV3101, *Sinorhizobium fredii* HH103. n =3 (One-way ANOVA with Tukey's multiple comparisons test Columns bearing different letters are significantly different.) Dimethyl phthalate (DP); Camphene (Cam); 3-FurAldehyde (Fur); (E)-2-Heptenal (eHep); Isobutyl butyrate (BuA); 1-Acetylimidazole (Ace).


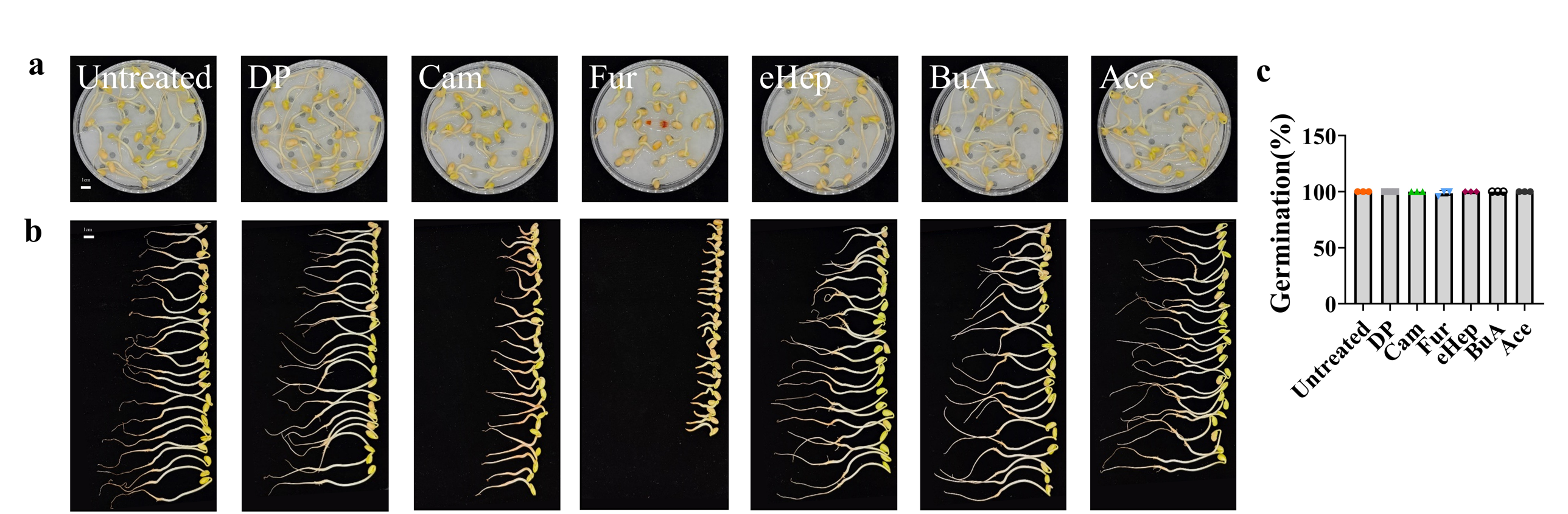


Extended Data Fig.5 Effect of volatile compounds (VOCs) on soybean DN50 seed germination and seedling growth. **a**, Effect of VOCs on DN50 germination for 4 days. **b**, Growth of DN50 sprouted for 5 days with VOCs. **c**, Statistics of VOCs on ear germination rate. n =24 (One-way ANOVA with Tukey's multiple comparisons test Columns bearing different letters are significantly different.) Dimethyl phthalate (DP); Camphene (Cam); 3-FurAldehyde (Fur); (E)-2-Heptenal (eHep); Isobutyl butyrate (BuA); 1-Acetylimidazole (Ace).


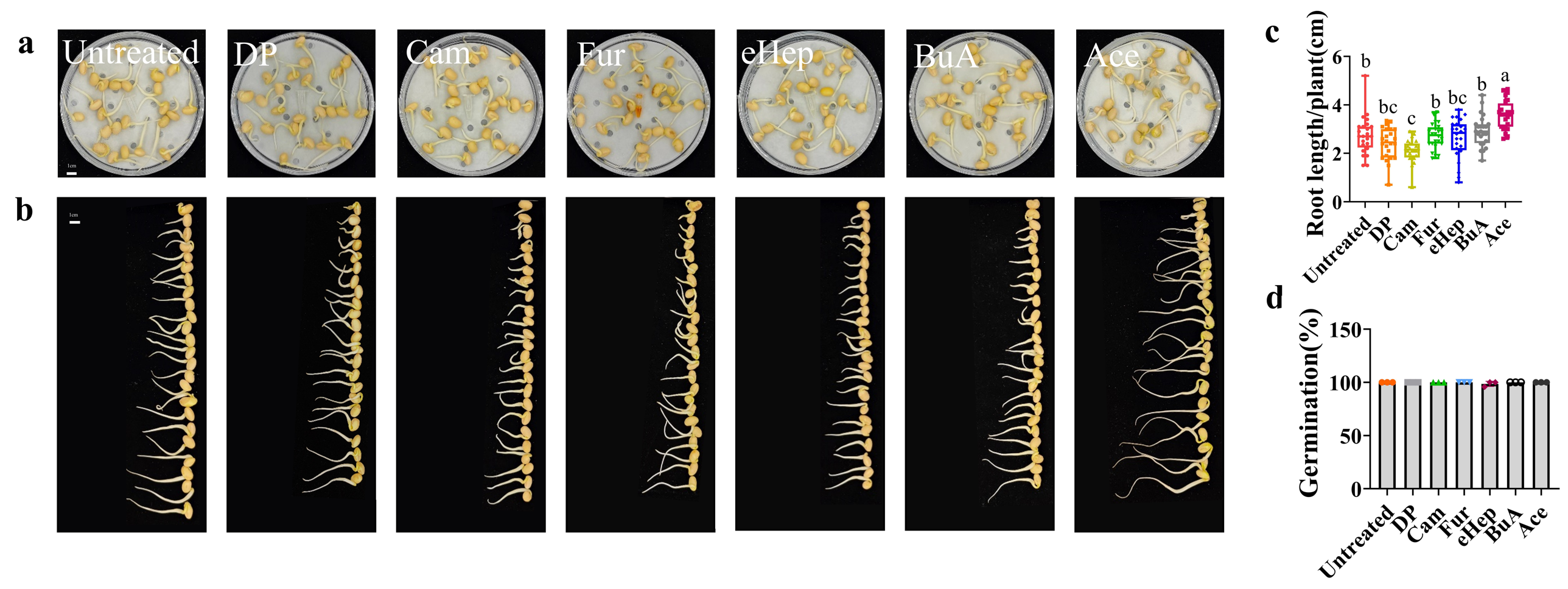


Extended Data Fig.6 Effect of volatile compounds (VOCs) on soybean SN14 seed germination and seedling growth. **a**, Effect of VOCs on SN14 germination for 4 days. **b**, Growth of SN14 sprouted for 5 days with VOCs. **c** Root length statistics of SN14 sprouted for 5 days with VOCs. n =24 **d,** Statistics of VOCs on ear germination rate. n =24 (One-way ANOVA with Tukey's multiple comparisons test Columns bearing different letters are significantly different.) Dimethyl phthalate (DP); Camphene (Cam); 3-FurAldehyde (Fur); (E)-2-Heptenal (eHep); Isobutyl butyrate (BuA); 1-Acetylimidazole (Ace).


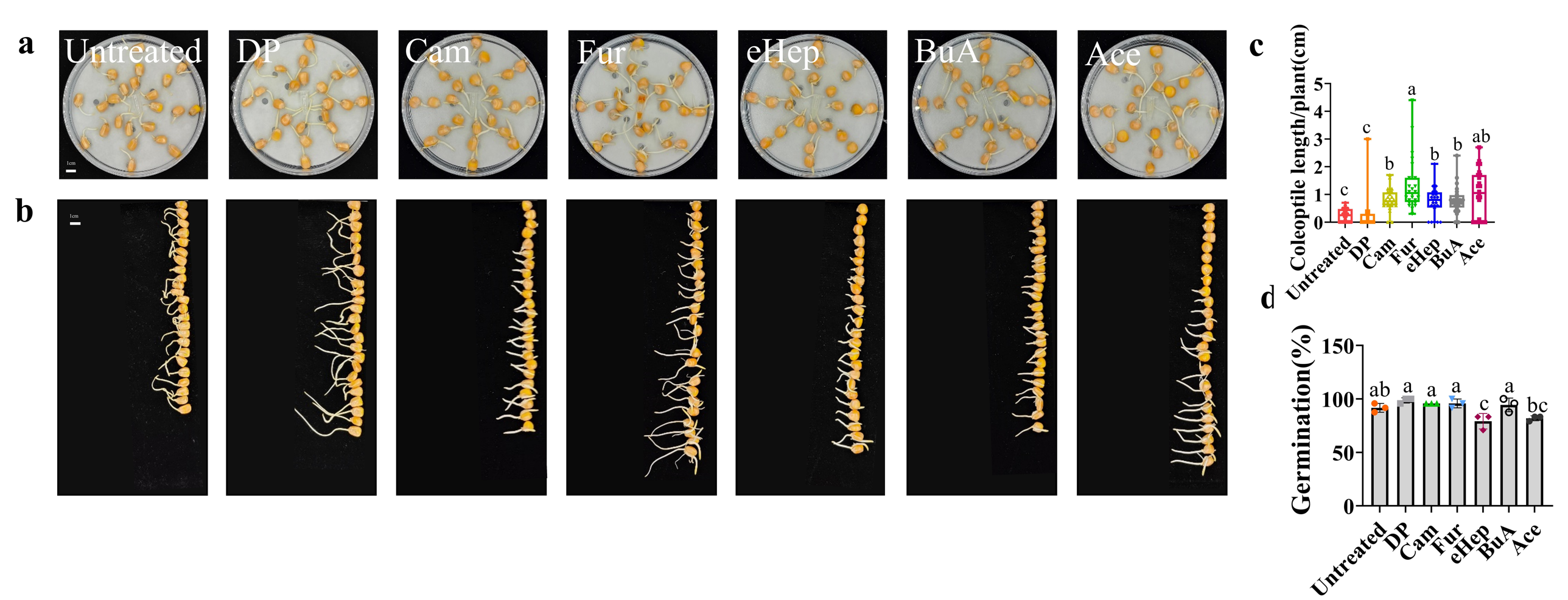


Extended Data Fig.7 Effect of volatile compounds (VOCs) on maize DN285 seed germination and seedling growth. **a**, Effect of VOCs on DN285 germination for 4 days. **b**, Growth of DN285 sprouted for 5 days with VOCs. **c**, Coleoptile length statistics of DN285 sprouted for 5 days with VOCs. n =24 **d**, Statistics of VOCs on ear germination rate. n =24 (One-way ANOVA with Tukey's multiple comparisons test Columns bearing different letters are significantly different.) Dimethyl phthalate (DP); Camphene (Cam); 3-FurAldehyde (Fur); (E)-2-Heptenal (eHep); Isobutyl butyrate (BuA); 1-Acetylimidazole (Ace).


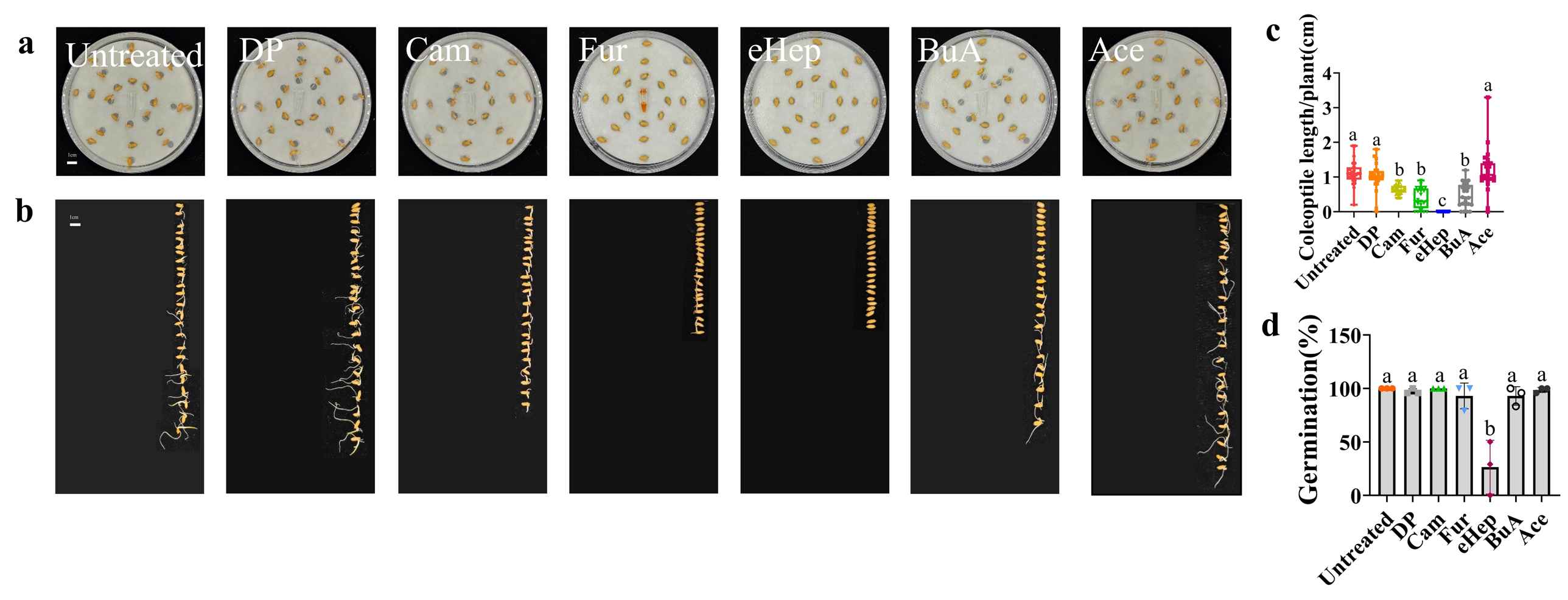


Extended Data Fig.8 Effect of volatile compounds (VOCs) on rice DN428 seed germination and seedling growth. **a**, Effect of VOCs on DN428 germination for 4 days. **b**, Growth of DN428 sprouted for 5 days with VOCs. **c**, Coleoptile length statistics of DN428 sprouted for 5 days with VOCs. n =24 **d**, Statistics of VOCs on ear germination rate. n =24 (One-way ANOVA with Tukey's multiple comparisons test Columns bearing different letters are significantly different.) Dimethyl phthalate (DP); Camphene (Cam); 3-FurAldehyde (Fur); (E)-2-Heptenal (eHep); Isobutyl butyrate (BuA); 1-Acetylimidazole (Ace).


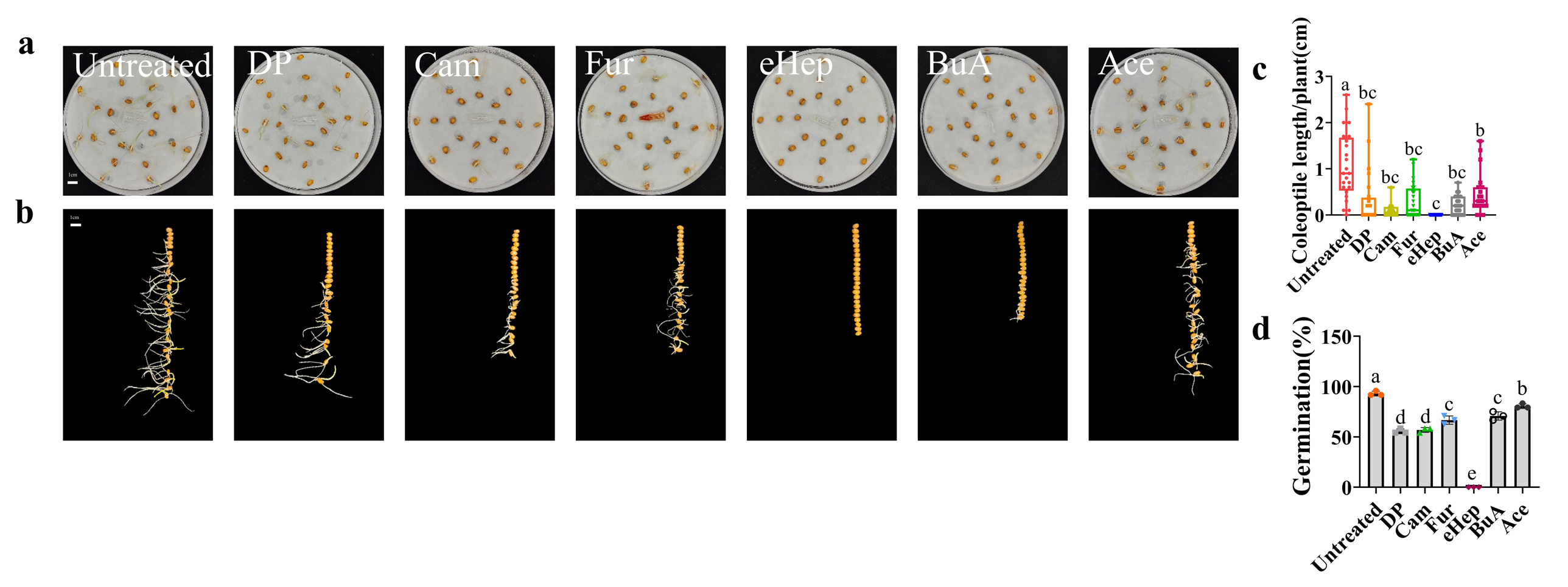


Extended Data Fig.9 Effect of volatile compounds (VOCs) on wheat DN127 seed germination and seedling growth. **a**, Effect of VOCs on wheat DN127 germination for 4 days. **b**, Growth of DN127 sprouted for 5 days with VOCs. **c**, Coleoptile length statistics of DN127 sprouted for 5 days with VOCs. n =24 **d**, Statistics of VOCs on ear germination rate. n =24 (One-way ANOVA with Tukey's multiple comparisons test Columns bearing different letters are significantly different.) Dimethyl phthalate (DP); Camphene (Cam); 3-FurAldehyde (Fur); (E)-2-Heptenal (eHep); Isobutyl butyrate (BuA); 1-Acetylimidazole (Ace).


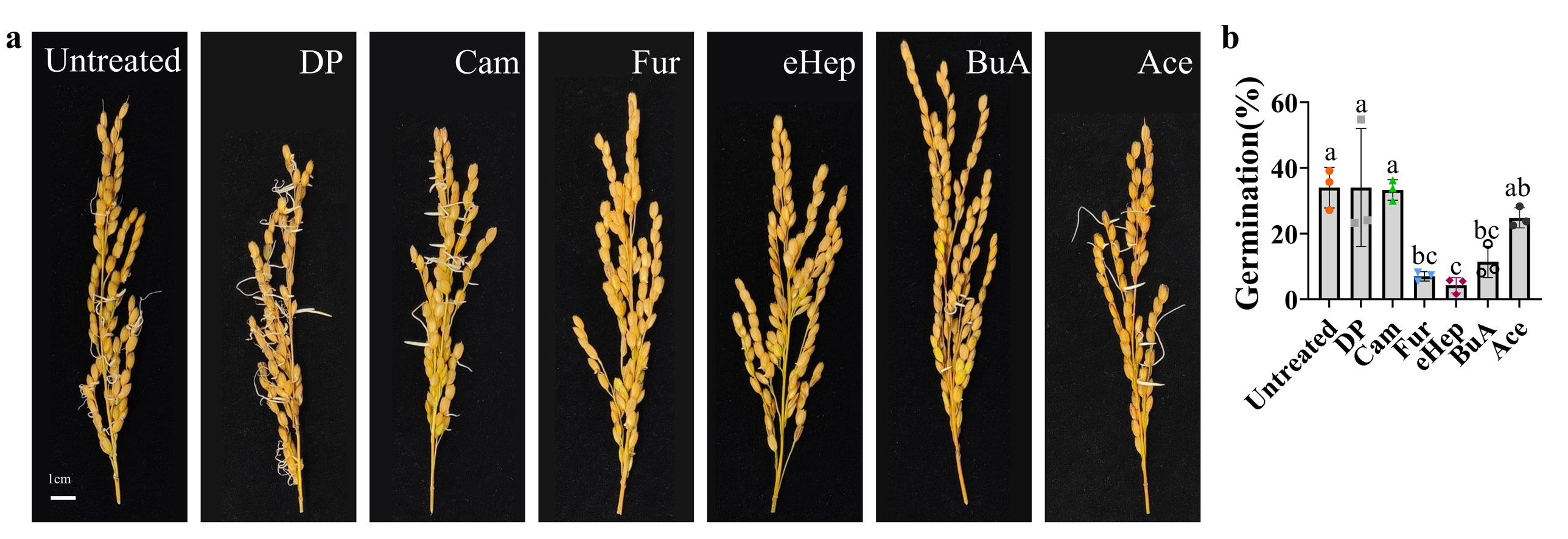


Extended Data Fig.10 Rice spike sprouting. **a**, Effect of volatile compounds (VOCs) on germination for 7 days. **b**, Statistics of VOCs on ear germination rate. Calculate the germination rate of rice seeds per panicle. n =3 (One-way ANOVA with Tukey's multiple comparisons test Columns bearing different letters are significantly different.) Dimethyl phthalate (DP); Camphene (Cam); 3-FurAldehyde (Fur); (E)-2-Heptenal (eHep); Isobutyl butyrate (BuA); 1-Acetylimidazole (Ace).
